# Molecular interplay between TXNIP and GLUT9 underlies uric acid transport dysregulation in vitro under hyperuricemic stress

**DOI:** 10.1186/s40001-025-03209-8

**Published:** 2025-10-09

**Authors:** Yuqiu Zhu, Bei Zhang, Yi He, Manxi Du, Wujin Chen, Xiaoyu Chen, Qingqing Yang, Mayina Kahaer, Yuping Sun

**Affiliations:** 1 Microbiology Department, Basic Medical College of Xinjiang Medical University, Urumqi, 830017 China; 2 Key Laboratory of Molecular Biology of Endemic Diseases, Xinjiang Uygur Autonomous Region, Urumqi, 830017 China; 3 Morphological Center, Basic Medical College of Xinjiang Medical University, Urumqi, 830017 China; 4 Department of Basic Medicine, Xinjiang Second Medical College, Karamay, 834000 China

**Keywords:** TXNIP, GLUT9, Hyperuricemia, HK-2 cells, Uric acid transport

## Abstract

**Background:**

This study aimed to investigate the role of Thioredoxin Interacting Protein (TXNIP) in hyperuricemia, specifically examining its influence on uric acid (UA) metabolism and the associated processes.

**Methods:**

A hyperuricemia cellular model was created using human renal tubular epithelial cells (HK-2). The DCFH-DA probe was utilized to quantify the quantities of reactive oxygen species (ROS) within cells. Cell lines exhibiting TXNIP overexpression and knockdown were established. The impact of TXNIP overexpression on UA metabolism in HK-2 cells was examined using integrated metabolomic and transcriptome analysis. Real-time fluorescence quantitative PCR (RT-qPCR) and Western blot were employed to assess the expression levels of TXNIP and the UA transporters GLUT9, ABCG2, URAT1, and OAT3. The connection between TXNIP and GLUT9 was investigated by the Co-immunoprecipitation. Additionally, immunofluorescence labeling was utilized to examine the subcellular localization of TXNIP and GLUT9.

**Results:**

A high UA environment stimulated ROS generation and markedly elevated TXNIP expression. Research utilizing metabolomics and transcriptomics suggests that TXNIP may modify the UA metabolic pathway in HK-2 cells, hence affecting UA stability. Conversely, TXNIP overexpression enhanced net UA uptake in HK-2 cells by elevating GLUT9 expression, but TXNIP knockdown yielded the contrary effect. Immunofluorescence labeling demonstrated the co-localization of TXNIP and GLUT9 in HK-2 cells. CO-IP studies demonstrated that TXNIP interacted with GLUT9.

**Conclusion:**

TXNIP may influence UA reabsorption in hyperuricemia via ROS-induced upregulation and subsequent enhancement of its expression through interaction with the UA transporter protein, GLUT9, hence impacting the progression of hyperuricemia.

**Supplementary Information:**

The online version contains supplementary material available at 10.1186/s40001-025-03209-8.

## Introduction

TXNIP (Thioredoxin Interacting Protein) was initially discovered in the human leukemia cell line HL60 following stimulation with 1,25-dihydroxyvitamin D3. TXNIP associates with thioredoxin (TRX) and impedes its function, therefore contributing to the regulation of redox equilibrium [1]. Hyperuricemia and gout are prevalent metabolic disorders, with their incidence rising annually in China [2]. Their principal characteristics encompass elevated blood uric acid (UA) levels, with the underlying pathophysiology closely linked to inflammatory responses [3, 4]. As hyperuricemia progresses to gout, UA crystals accumulate in joints and other tissues, eliciting a localized inflammatory response that results in pain, erythema, and edema. Inflammatory cells release several inflammatory mediators, such as IL-1β, IL-6, and TNF-α, which exacerbate the inflammatory response [5]. Research on the impact of TXNIP on hyperuricemia and gout is progressively expanding annually. Inflammatory vesicle activators, such as UA, can activate NLRP3 inflammasomes by inducing the dissociation of TXNIP from the TXNIP/TRX complex, thereafter binding to NLRP3 in a Reactive oxygen species (ROS)-dependent way [6–8]. Furthermore, TXNIP can augment the inflammatory response by stimulating the MAPK (Mitogen-Activated Protein Kinase) and NF-κB (Nuclear Factor-κB) signaling pathways [9–12]. Consequently, TXNIP is an essential target in the investigation of hyperuricemia and gout. Although TXNIP's role in inflammation associated with hyperuricemia and gout has been widely recognized as a key regulator of oxidative stress, its involvement in UA metabolism remains unconfirmed. Previous studies indicate TXNIP plays a crucial role in regulating glucose metabolism; for instance, it inhibits cellular glucose uptake by reducing GLUT1 expression and inducing GLUT1 internalization [13, 14]. Studies have also shown that during the in vitro maturation of bovine oocytes, TXNIP influences the expression of key enzymes in glycolysis, such as PFK and G6PDH, potentially affecting glucose metabolism by regulating their expression [15]. Knocking out TXNIP not only alleviates diabetic kidney fibrosis but also improves cardiac function in obese mice [16, 17]. Furthermore, proteomics studies have demonstrated that TXNIP expression is significantly elevated in rats with chronic kidney disease [18]. Hyperuricemia and gout are diseases caused by impaired UA metabolism, which can further trigger inflammation and kidney damage. They are closely associated with metabolic disorders such as diabetes and obesity [19, 20]. Therefore, as an important target in metabolic syndrome, the role of TXNIP in UA metabolism warrants further investigation. In this study, we propose the hypothesis that TXNIP regulates UA transport by modulating GLUT9 expression in HK-2 cells, based on the integration of metabolomics and transcriptomics analyses. Our in vitro experiments indicate that TXNIP influences UA reabsorption through its interaction with GLUT9. This novel finding contributes to a more comprehensive understanding of the mechanisms underlying TXNIP's role in UA metabolism and offers a novel theoretical framework for the treatment of hyperuricemia and gout.

## Results

### High levels of UA induce ROS production and upregulate TXNIP

**Fig. 1:**
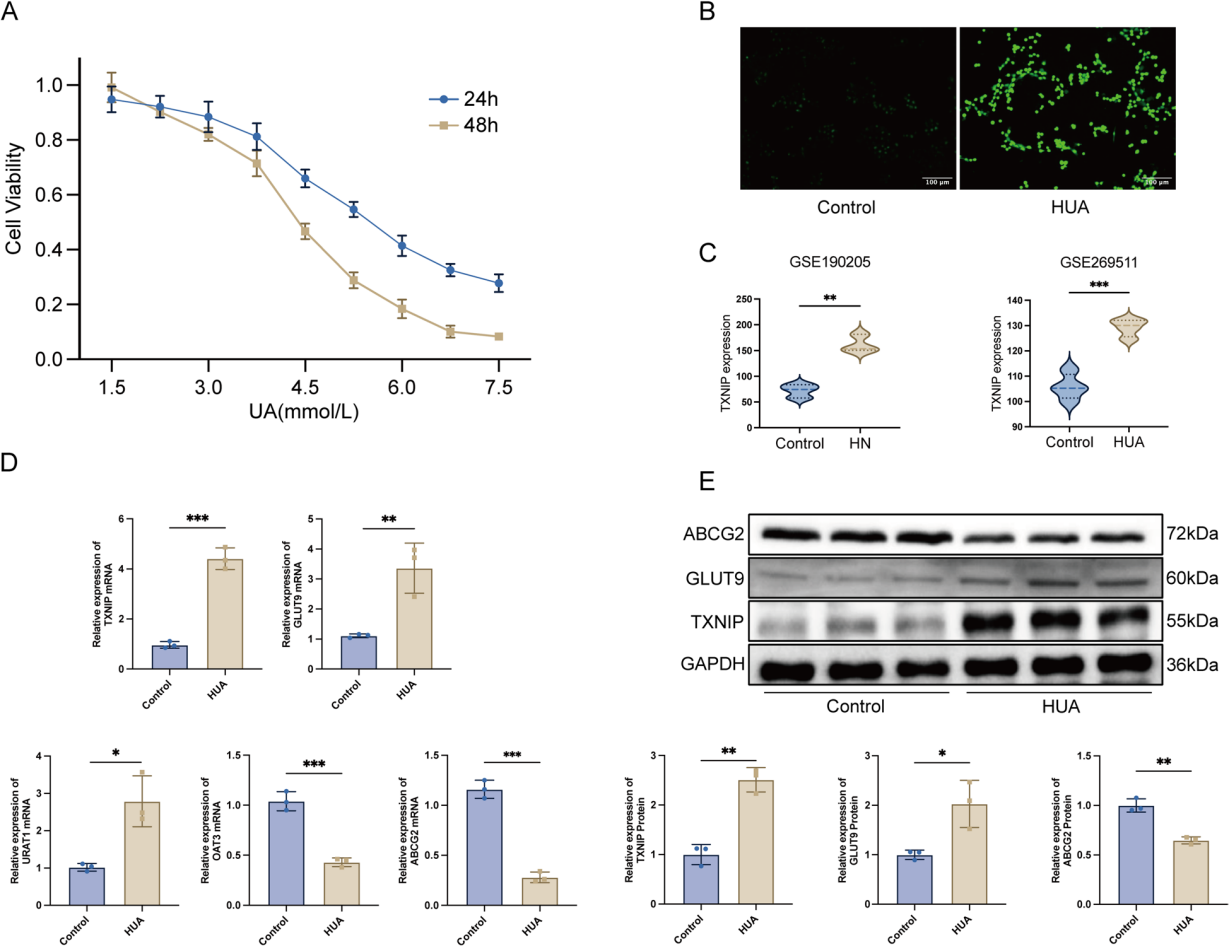
TXNIP is highly expressed in the model of hyperuricemia.** A** CCK-8 assay (*n* = 3); **B** Representative immunofluorescence micrographs of DCFH-DA detects the level of ROS levels within HK-2 cells (*n* = 3); **C** TXNIP mRNA levels were explored in the GEO database, with TXNIP levels compared between healthy mice (Control) (*n* = 3) and hyperuricemic nephropathy mice (HN) (*n* = 3), and also between healthy mice (Control) (*n* = 4) and hyperuricemic mice (HUA) (*n* = 4); **D** RT-qPCR detects the mRNA level expression of TXNIP, GLUT9, URAT1, ABCG2 and OAT3 (*n* = 3);** E** Western blot detects the protein level expression of TXNIP and GLUT9, and ABCG2 (*n* = 3). Data were displayed as mean ± SEM. * indicates comparison with the control group, *P < 0.05, **P < 0.01, ***P < 0.001

HK-2 cells were subjected to varying doses of UA. The CCK-8 results (Fig. 1A) indicated that cell viability progressively diminished with elevated UA concentration and prolonged treatment Duration. At 48 h, cell viability markedly decreased with increasing UA content. At 24 h, when the UA concentration was beyond 3.75 mmol/L, cell viability exhibited a considerable alteration, although it remained above 80% at concentrations equal to or below 3.75 mmol/L. According to these findings, the UA stimulation Duration was established at 24 h, with a concentration of 3.75 mmol/L for the hyperuricemia (HUA) group intervention in this investigation. The DCFH-DA probe assay results indicated a significant increase in intracellular ROS levels in the HUA group compared to the control group (Fig. 1B). Simultaneously, our analysis of renal transcriptome data from mice with hyperuricemia and hyperuricemic nephropathy (HN), as available in the GEO database, revealed a significant upregulation of TXNIP at the transcriptional level compared to the control group (Fig. 1C). This finding indicates that TXNIP may play a crucial role in the pathogenesis of hyperuricemia and its associated disorders. The results of our real-time quantitative polymerase chain reaction (RT-qPCR) analysis corroborated the aforementioned findings. Specifically, we observed a significant elevation in the mRNA levels of TXNIP in HK-2 cells within the HUA group. Additionally, there was an upregulation in the mRNA expression levels of the uric acid reabsorption proteins, GLUT9 and URAT1. In contrast, the mRNA expression levels of the uric acid excretion proteins, ABCG2 and OAT3, were downregulated (Fig. 1D). The findings of the Western blotting indicated a considerable increase in TXNIP and GLUT9 protein levels in the HUA group, but ABCG2 exhibited a decrease (Fig. 1E). The initial findings indicate that elevated UA compensatorily enhances UA reabsorption and reduces its excretion in HK-2 cells. Furthermore, it serves as a catalyst to promote ROS generation and enhance TXNIP expression.

### TXNIP overexpression affects UA metabolism in HK-2 cells

**Fig. 2:**
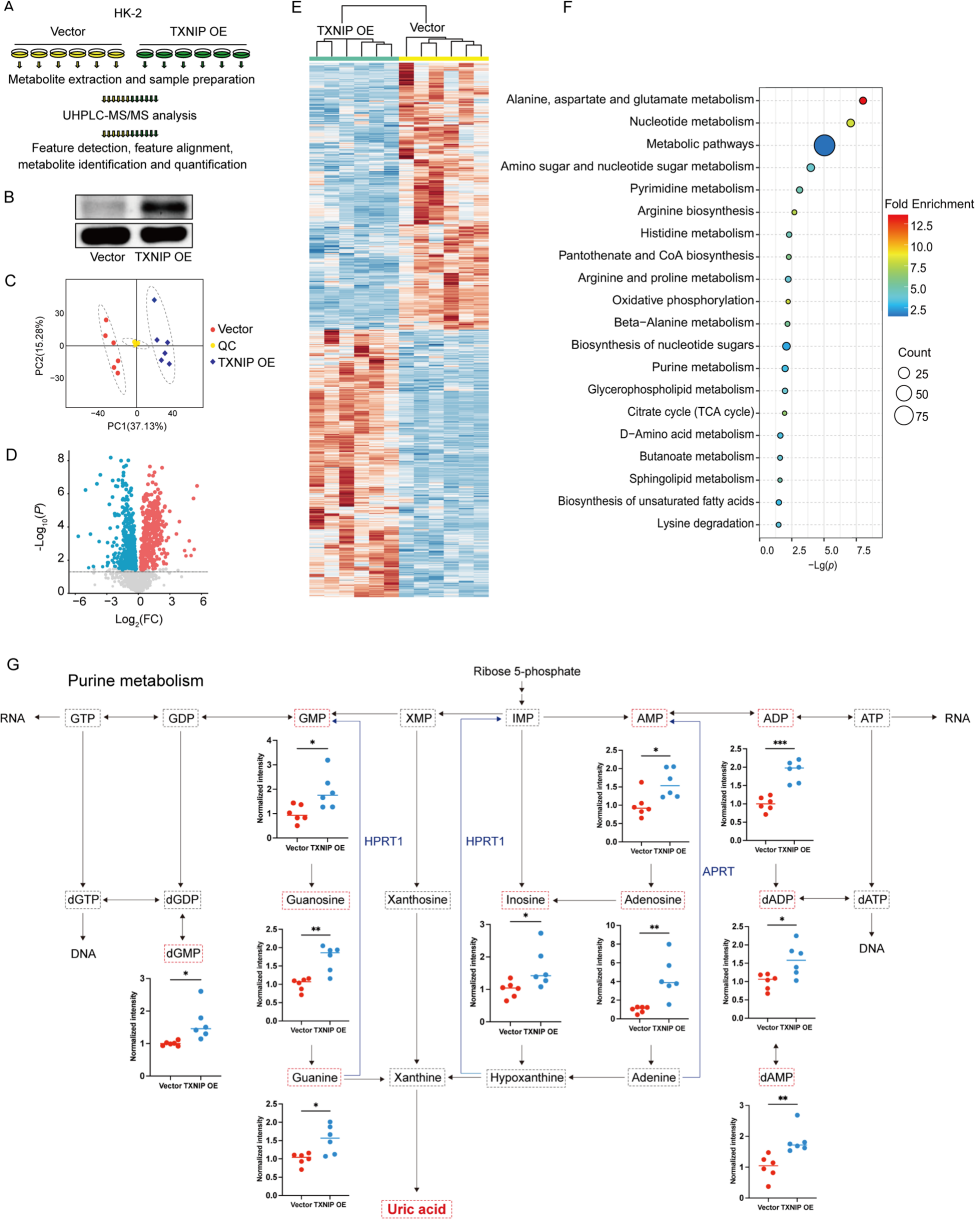
HK-2 cell UA metabolism has changed after TXNIP overexpression.** A** The untargeted metabolome analysis process was performed on the Vector (*n* = 6) and TXNIP OE (*n* = 6) groups; **B** Western blot showing TXNIP protein expression levels in HK-2 cells of Vector (*n* = 3) and TXNIP OE (*n* = 3) groups; **C**: Principal Component Analysis (PCA) 3D score plots of metabolomics data; **D** Volcano plots of significant changes in all detected metabolites (Up-regulated metabolites are indicated by red dots, down-regulated metabolites are indicated by blue dots); **E** Hierarchical clustering of differential metabolites; **F** KEGG pathway enrichment map; **G** Relative normalized levels of metabolites in the Purine metabolism. Data were displayed as mean ± SEM. * indicates comparison with the Vector group, *P < 0.05, **P < 0.01, ***P < 0.001

To clarify the mechanism of TXNIP action at elevated UA circumstances, we developed a stable HK-2 cell line that overexpresses TXNIP. Lentiviral transduction facilitated this achievement. Comparative metabolomic analyses were performed between the Vector group and the TXNIP OE group to detect, measure, and characterize different metabolites (Fig. 2A). Western blot analysis validated successful TXNIP overexpression (Fig. 2B), revealing markedly elevated TXNIP protein levels in the TXNIP OE group (*P* < 0.001). Metabolomic analysis demonstrated that TXNIP overexpression caused major alterations in the HK-2 cell metabolome: 434 metabolites were markedly elevated, 417 were notably downregulated, and 1,003 exhibited no significant variation (*P* < 0.05) (Fig. 2D). Principal component analysis (PCA) indicated clear clustering between the TXNIP overexpression and vector control groups (Fig. 2C), whilst Hierarchical Clustering exhibited metabolite signatures particular to each group (Fig. 2E). These approaches illustrate the technical reproducibility and biological relevance of the results. KEGG pathway enrichment analysis revealed that the metabolic pathways affected by TXNIP overexpression were considerably enriched in alanine, aspartate, and glutamate metabolism, nucleotide metabolism, purine metabolism, and the citrate cycle (TCA cycle) (Fig. 2F). Aspartic acid serves as a crucial precursor in purine nucleotide synthesis, which is vital for purine biosynthesis and directly influences UA generation. The TCA cycle serves as the primary center for cellular energy (ATP) generation and carbon skeleton provision. Its modification immediately indicates the changes of cellular energy metabolic condition. Purine metabolism directly impacts UA generation, and modifications in these pathways due to TXNIP overexpression influence cellular energy metabolism and the advancement of metabolic syndrome via systemic regulation. Subsequently, we conducted an enrichment study of Primary Pathways from SMPDB, a subset of the Human Metabolic Database (HMDB). The findings indicated that differential metabolites were markedly enriched in Pantothenate and CoA Biosynthesis (Fig. S1), whereas the breakdown of CoA and its sulfate production serve a crucial physiological function in enhancing renal UA excretion. Pantothenic acid, a precursor of CoA, indirectly facilitates this process by aiding in CoA production. Consequently, TXNIP evidently plays a significant role in the metabolism of HK-2 cells. Normalization analysis results demonstrated that TXNIP overexpression markedly elevated critical precursors in the UA synthesis pathway, including Guanine, Guanosine, GMP, and Adenosine (Fig. 2G). The elevated levels of these purine-related metabolites indicate a potential correlation between TXNIP and the overproduction of UA. In conclusion, TXNIP overexpression modified UA metabolism in HK-2 cells.

### The transportation of UA in HK-2 cells is affected by TXNIP overexpression

**Fig. 3:**
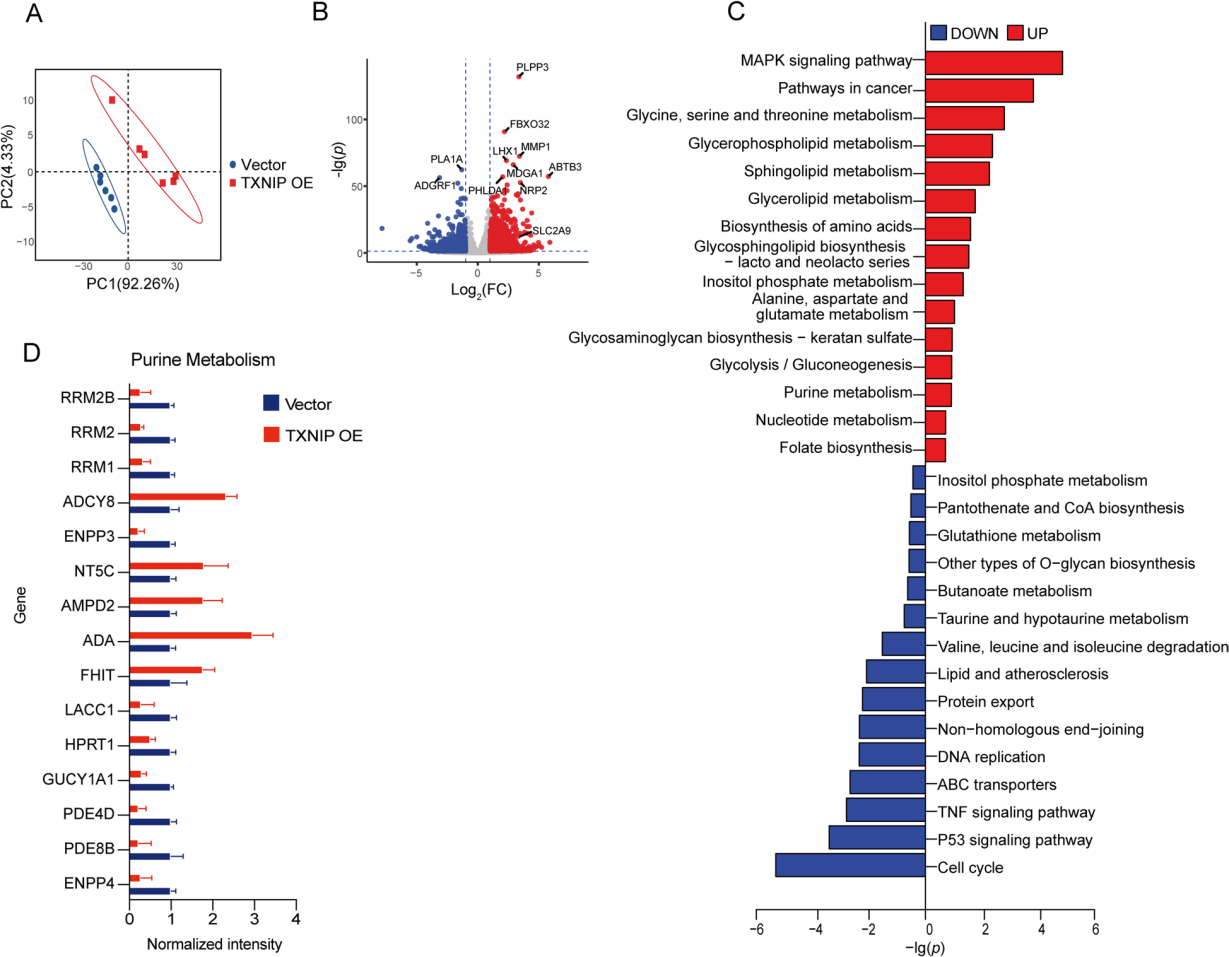
TXNIP overexpression affects UA transport in HK-2 cells. **A** Transcriptomics analysis was performed on the Vector (*n* = 6) and TXNIP OE (*n* = 6) groups. A PCA plot of the transcriptomics data was created; **B** Volcano plot of DEGs. (Red dots indicates up-regulated DEGs, blue dots indicates down-regulated DEGs); **C** KEGG pathway enrichment results; **D** Normalized expression of key genes related to Purine metabolism. Data were displayed as mean ± SEM

To verify that TXNIP modulates UA metabolism, we subsequently conducted transcriptome profiling on HK-2 cells from both the Vector and TXNIP OE groups. High-quality transcriptome data demonstrated significant similarity among samples within each group and a distinct split between the two groups (Fig. 3A). Using the criterion of Log_2_ FC > 1 or Log_2_ FC < − 1 in conjunction with* P* < 0.05, the DEGs between the Vector and TXNIP OE groups were identified. A total of 3432 DEGs were identified, comprising 1566 up-regulated genes and 1866 down-regulated genes. Notably, there is a substantial up-regulation of *SLC2A9*, a gene that encodes the UA reabsorption protein GLUT9, indicating that TXNIP may influence UA metabolism (Fig. 3B). Gene Ontology functional annotation and enrichment analysis (Fig. S2) indicated that DEGs were considerably enriched in metabolic pathways, signaling pathways, and cell cycle control within biological processes. Simultaneously, the DEGs were mostly situated in particular organelles, cell membranes, and macromolecular complex areas, and in terms of molecular function categories, they exhibited considerable enrichment in binding and catalytic activity. The findings of this section further corroborate our assertion that TXNIP is a crucial factor in UA metabolism.

Utilizing the KEGG database, we conducted pathway enrichment analysis of the differentially expressed genes. This research revealed 15 pathways that were considerably up-regulated and 15 pathways that were significantly down-regulated subsequent to TXNIP overexpression. The upregulated pathways encompassed the MAPK signaling route, glycine, serine, and threonine metabolism pathway, purine metabolism, nucleotide metabolism, among others (Fig. 3C). Glycine and Serine, together with their derived one-carbon units from folate, are essential precursor substrates in the de novo synthesis route of purine nucleotides. Glycine, serine, and threonine metabolism are intricately associated with amino acid metabolism and strongly interconnected with purine metabolism, collectively governing cellular function and metabolic equilibrium. KEGG analysis indicated that some genes associated with purine metabolism and UA precursor synthesis were upregulated following TXNIP overexpression. Furthermore, a significant level of concordance was noted between the transcriptome and metabolomic results. Notably, we observed that the expression of the ABC transporter gene set, linked to UA transport, was down-regulated (Fig. 3C). Furthermore, the normalization of genes associated with purine metabolism indicated that TXNIP overexpression led to an upregulation of ADA and a downregulation of HPRT1 (Fig. 3D). ADA facilitates a crucial process in purine metabolism by irreversibly deaminating adenosine to inosine and deoxyadenosine to deoxyinosine. It is an essential enzyme in the degradation of purine nucleotides. HPRT1 is a key enzyme in the purine salvage system, whose decreased expression may relieve route obstruction, consequently altering purine base and UA buildup. We anticipated that the overexpression of TXNIP could impact the UA cycle in HK-2 cells by affecting the synthesis of UA precursors and UA transport.

### TXNIP affects the expression of UA reabsorption protein GLUT9

**Fig. 4:**
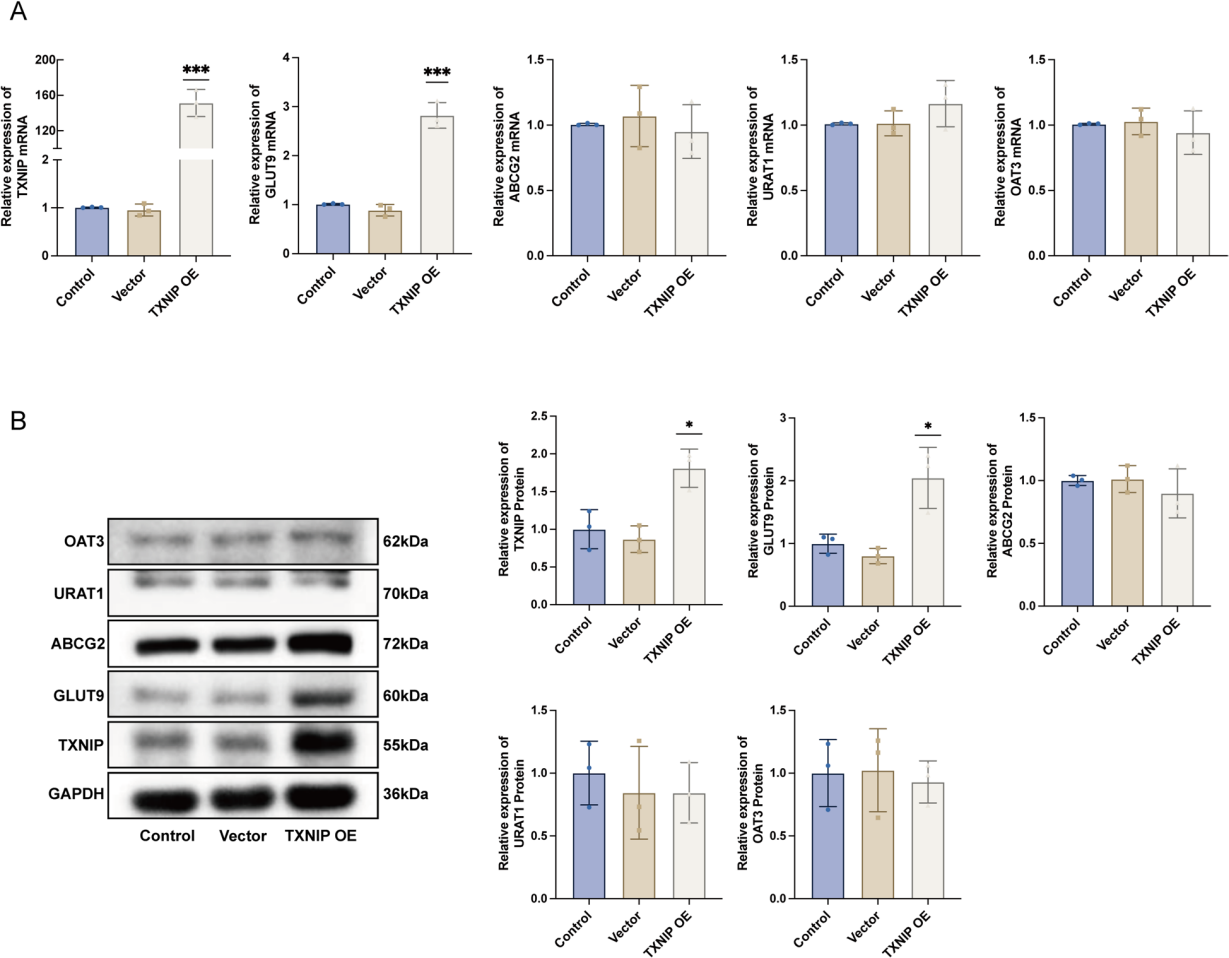
TXNIP affects the expression of GLUT9. **A** RT-qPCR to detect the mRNA level expression of TXNIP and GLUT9, URAT1, ABCG2, OAT3 (*n* = 3); **B** Western blot to detect the protein level expression of TXNIP and GLUT9, URAT1, ABCG2, OAT3 (*n* = 3). Data were displayed as mean ± SEM. * indicates comparison with the Control group, *P < 0.05, ***P < 0.001

The combined analysis of the metabolome and transcriptome prompted us to explore the regulatory role of TXNIP on UA transport proteins. Consequently, we undertook further studies to investigate this possibility. In the TXNIP OE group, RT-qPCR results indicated that TXNIP and GLUT9 mRNA levels were markedly elevated compared to the control group. In contrast, the mRNA expression levels of URAT1, ABCG2, and OAT3 exhibited relative stability, with no significant changes detected (Fig. 4A). The Western blot analysis demonstrated a significant elevation of TXNIP and GLUT9 protein levels in the TXNIP overexpression group compared to the control group. Conversely, there was no significant alteration in URAT1, ABCG2, and OAT3 (Fig. 4B). The aforementioned data tentatively indicate that TXNIP may modulate the expression of GLUT9.

### TXNIP regulates UA transport by modulating GLUT9 expression

**Fig. 5:**
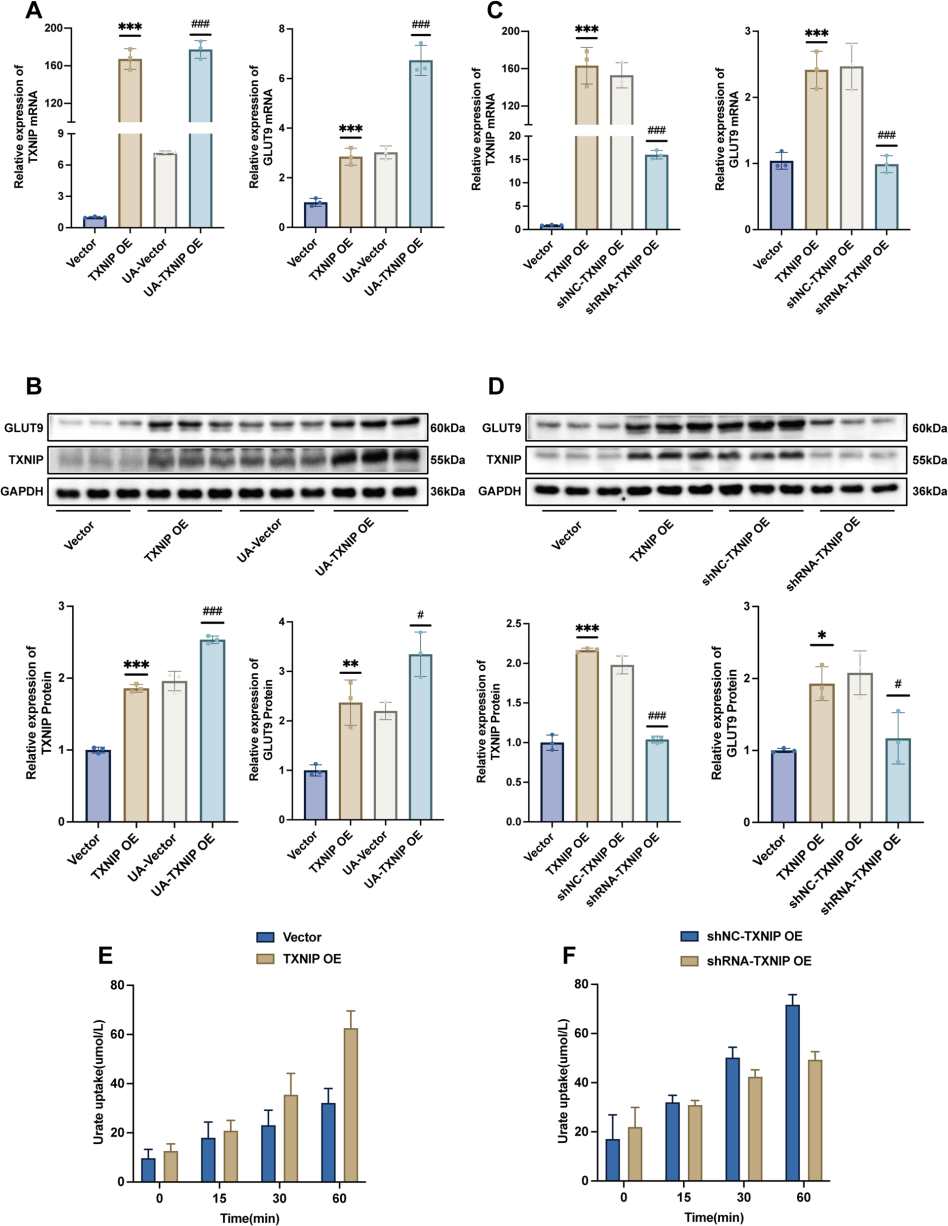
TXNIP regulates UA transport by modulating GLUT9. **A** and **C** RT-qPCR for mRNA levels of TXNIP and GLUT9 (*n* = 3); **B** and **D** Western blot for protein levels of TXNIP and GLUT9 (*n* = 3);** E** and **F** Effect of overexpression/knockdown of TXNIP on net UA uptake in HK-2 cells (*n* = 3). Data were displayed as mean ± SEM. * indicates comparison with the Vector group, ^#^ indicates comparison with the UA-Vector/shNC-TXNIP OE group, *^/#^*P* < 0.05, **P < 0.01, ***^/###^*P* < 0.05

To further examine the putative mechanism by which TXNIP regulates GLUT9, we then performed cellular tests. The results demonstrated that in HK-2 cells with stable TXNIP overexpression exposed to enhanced UA circumstances, the mRNA (Fig. 5A) and protein (Fig. 5B) levels of TXNIP and GLUT9 were significantly increased. In HK-2 cells with stable TXNIP overexpression, TXNIP knockdown resulted in a significant reduction of mRNA (Fig. 5C) and protein (Fig. 5D) levels of both TXNIP and GLUT9 in the shRNA group compared to the shNC group. This indicates that TXNIP modulates the expression of GLUT9. Simultaneously, we observed that following treatment with an identical concentration of UA for the same duration, the net UA uptake in HK-2 cells of the TXNIP OE group exceeded that of the Vector group, while TXNIP knockdown led to a lesser increase in net UA uptake compared to the shNC-TXNIP OE group. In accordance with the anticipated outcomes, TXNIP governed UA transport by influencing GLUT9 expression.

### TXNIP interacts with GLUT9 in HK-2 cells

**Fig. 6:**
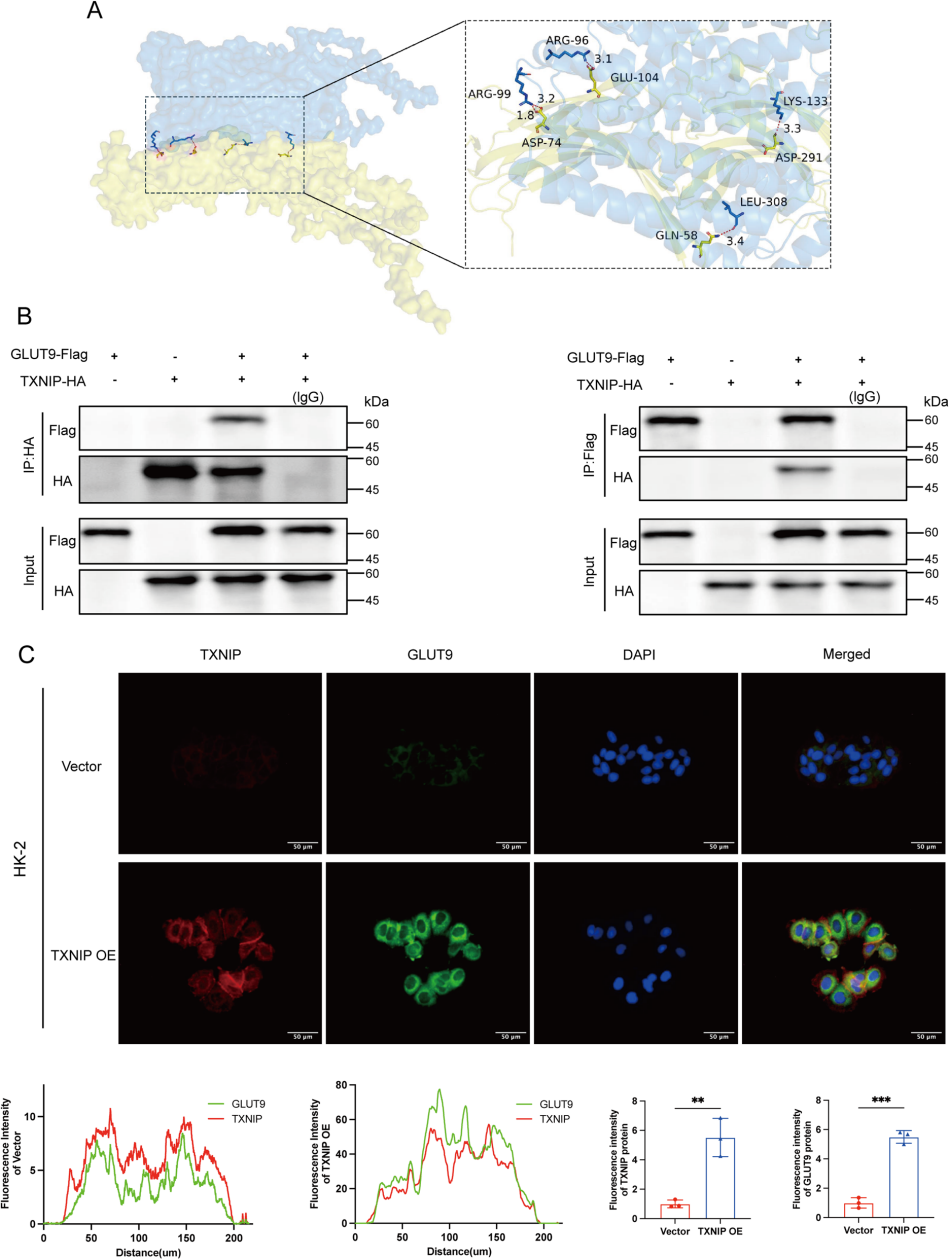
TXNIP interacts with GLUT9 in HK-2 cells. **A** Protein docking map (yellow for TXNIP and blue for GLUT9); **B** Co-IP in HEK 293 T cells using anti-HA antibody (left) or anti-Flag antibody (right); **C** Representative immunofluorescence micrographs of TXNIP and GLUT9 immunofluorescence in HK-2 cells (*n* = 3). Data were displayed as mean ± SEM. * indicates comparison with the Vector group, **P < 0.01, ***P < 0.001

To examine the precise mechanism by which TXNIP modulates GLUT9, we conducted protein–protein docking with GRAMM (Fig. 6A). The findings indicated that the interaction between TXNIP and GLUT9 was enhanced during the simulation, exhibiting a binding free energy of −18.7 kcal/mol (Table S3). Docking study revealed that TXNIP interacted with the GLUT9 surface, demonstrating substantial binding energy. Increased interaction sites between the two proteins, coupled with enhanced surface contacts, augmented complex stability via hydrogen bonding and improved binding stability. We performed co-immunoprecipitation in HEK293 cells overexpressing TXNIP-HA and GLUT9-Flag to investigate the physical connection between TXNIP and GLUT9, confirming their association (Fig. 6B). The TXNIP-HA protein precipitated the GLUT9-Flag protein in HEK293 cells. Similarly, a GLUT9- Flag complexed with TXNIP-HA in the co-immunoprecipitation experimental conditions. Immunofluorescence demonstrated that the fluorescent expression of GLUT9 in HK-2 cells was elevated following the overexpression of TXNIP, hence corroborating our prior findings. TXNIP and GLUT9 were predominantly co-localized in the cytoplasm, with a minor presence in the nucleus (Fig. 6C).

## Methods

### Cell cultivation

HEK293 (Cat# STCC10305P-1, Servicebio) cells and Human renal tubular epithelial cells (HK-2 cells) (Cat# CL-0109, Procell) were cultivated in HEK293 Cell Complete Medium (Cat# GZ10305, Servicebio) and HK-2 Cell Complete Medium (Cat# CM-0109, Procell).

### Configuration and intervention of UA solutions

0.4 g of sodium hydroxide powder was dissolved in 9 ml of distilled water to make a sodium hydroxide solution. Subsequently, 0.1 g of UA (Cat# U820317, Macklin) powder was solubilized in 9 ml of sodium hydroxide solution. Concentrated hydrochloric acid was utilized to neutralize the solution's pH. Thereafter, distilled water was added to increase the solution's volume to 10 ml, resulting in a 10 mg/ml UA stock solution. The stock solution was diluted sequentially to the required concentrations using MEM basal medium. The control group received basal medium devoid of UA for 24 h, while the HUA group received a UA intervention concentration of 3.75 mmol/L for 24 h.

### CCK‑8 assays

HK-2 cells were enzymatically digested with trypsin, and cell enumeration was performed utilizing a hemocytometer. The HK-2 cells were grown with different doses of UA medium. After the incubation period, PBS was employed to wash the cells subsequent to the removal of the culture medium. Introduce the CCK8 reagent (Cat# CPD001S-BR5, Cytosci) and incubate as per the provided instructions. The optical density at 450 nm was subsequently evaluated utilizing a microplate reader.

### Intracellular ROS detection

ROS were identified utilizing the ROS Assay Kit (Cat# S0034S, Beyotime) with the DCFH-DA probe. Expose HK-2 cells from the control group and HUA group to a DCFH-DA solution at 37 °C for 20 min. Fluorescence was evaluated by confocal microscopy to analyze fluorescence expression.

### RT‑qPCR

Total cellular RNA was extracted utilizing the *TransZol* UP Plus RNA Kit (Cat# ER501, TransGen Biotech). The RNA was transcribed into complementary DNA (cDNA) utilizing ABScript II RT Master Mix for qPCR (Cat# RK20402, ABclonal). The cDNA was thereafter kept at −20 ℃. Primers for TXNIP, GLUT9, URAT1, ABCG2, OAT3, and β-actin were developed utilizing the NCBI database (Table S1). The qPCR reaction was subsequently conducted utilizing 2 × Universal SYBR Green Fast qPCR Mix (Cat# RK21203, ABclonal). The levels of mRNA were normalized to β-actin and measured using the 2^−ΔΔCT^ method.

### Western blot

Isolation of cellular proteins utilizing the Whole Protein Extraction Kit (Cat# BC3710, Solarbio). Combine the protein solution with 5 × loading buffer (Cat# P1040, Solarbio), then separate proteins of varying molecular weights using SDS-PAGE gel electrophoresis. Transfer the proteins from the gel to a PVDF membrane (Cat# IPVH00010, Millipore). The membranes were treated with TXNIP (Cat# 18243-1-AP, Proteintech), GLUT9 (Cat# 26486-1-AP, Proteintech), URAT1 (Cat# 14937-1-AP, Proteintech), ABCG2 (Cat# ab130244, Abcam), OAT3 (Cat# 16844-1-AP, Proteintech), and GAPDH (Cat# 60004-1-IG, Proteintech). On the second day, the membranes were treated with HRP-conjugated Goat Anti-Rabbit IgG (Cat# SA00001-2, Proteintech). Ultimately, protein bands were identified using a highly sensitive ECL chemiluminescent substrate (Cat# BL520A, Biosharp).

### Construction of lentiviral overexpression vectors and cell transfection

Lentivirus was acquired from Shanghai Jimma Pharmaceutical Technology Co., Ltd., and the vector type was LV5 (EF-1a/GFP&Puro), which includes the green fluorescent protein EGFP. The infection tests were divided into four categories: Control group (complete medium); Polybrene group (complete medium plus Polybrene); Vector group (complete medium plus control lentivirus and Polybrene); TXNIP OE group (complete medium plus TXNIP-overexpressing lentivirus and Polybrene). Cell morphology and viability were assessed 12–24 h post-infection to ascertain the necessity of medium replacement. Identical observations were recorded at 72 h post-infection, with medium alterations executed as necessary. Puromycin selection commenced 72 h post-infection and persisted until the control and Polybrene groups were entirely deceased. Monoclonal cell lines were subsequently obtained via the limiting dilution technique following the verification of fluorescence expression through confocal fluorescence microscopy.

### Construction of short hairpin RNA (shRNA) interference vectors and cell transfection

Interference target sites were developed based on the TXNIP gene transcript, and the matching primers were produced. The target sequences are included in Table S2. The interference plasmid (pGPU/GFP/Neo-shRNA) and the control plasmid (pGPU/GFP/Neo-shNC) were initially generated. Thereafter, these recombinant plasmids were introduced into E. coli DH5α. Positive transformants were tested and validated by sequencing. Utilize the Endotoxin-free Plasmid Medium Extraction Kit (Cat# DP108, TIANGEN) for the extraction of plasmid DNA. The transfection studies were categorized into four groups: Vector group; TXNIP OE group; shNC-TXNIP OE group (Transfectin g control plasmid); shRNA-TXNIP OE group (Transfecting interference plasmid). The transfection was performed using Lipofectamine 3000 (Cat# L3000015, Thermo Fisher Scientific) in accordance with the manufacturer's procedure.

### Untargeted metabolome sequencing and analysis

Metabolites were extracted from Vector group and TXNIP OE group for further LC–MS analysis. Additionally, to uphold data quality in metabolic profiling, quality control samples were generated by amalgamating sections of all samples that represent the complete set under analysis, which were utilized for data normalization. The preliminary mass spectrometry data was processed using MS-DIAL for peak alignment, retention time modification, and peak area extraction. Metabolites were discovered using precise mass measurements (mass tolerance < 10 ppm) and MS/MS data (mass tolerance < 0.02 Da) and subsequently matched with the HMDB public database. All multivariate data analyses and modeling in this work were conducted using R (version 4.0.3) along with the relevant R packages. We utilized the KEGG database (http://www.kegg.jp) to examine the pathways of the differential metabolites to discover the disturbed biological pathways. KEGG enrichment analyses were carried out with the Fisher’s exact test, and FDR correction for multiple testing was performed. The KEGG pathways with *P* < 0.05 were considered statistically significant. Metabolites are aligned with the HMDB public database.

### Transcriptome sequencing and analysis

After extracting total RNA from the Vector group and the TXNIP OE group, its concentration and integrity were assessed utilizing a NanoDrop spectrophotometer (Thermo Scientific). Sequencing Libraries were generated from 3 μg of RNA. The sequencing library was processed on the NovaSeq Xplus platform (Illumina), producing raw data in FASTQ format. HTSeq (0.9.1) was utilized to quantify gene expression by calculating the read count for each gene, hence determining the initial expression level. The values were subsequently adjusted using FPKM. Differentially Expressed Genes (DEGs) were evaluated using DESeq (1.30.0) with the criteria of |log_2_FoldChange|> 1 and a significance threshold of *P* < 0.05. Using topGO to perform GO enrichment analysis on the DEGs, calculate *P*-value by hypergeometric distribution method (the standard of significant enrichment is *P* < 0.05). Conduct KEGG pathway enrichment analysis for DEGs using ClusterProfiler (version 3.4.4), concentrating on pathways that exhibited substantial enrichment (*P* < 0.05).

### Intracellular uric acid assay

The original growth media was removed from the HK-2 cells, which were then cultivated in a medium enriched with 100 µmol/L UA. After incubation periods of 0, 15, 30, and 60 min, the media containing UA was discarded, and the cells were rinsed with a PBS solution. Subsequently, cells were harvested at each time point, and cellular UA levels were quantified following the protocol of the Amplex Red UA & Uricase Test Kit (Cat# S0231S, Beyotime). The fluorescence value in the cell lysate was utilized to indicate the intracellular UA level, comprising the baseline intracellular UA content and the quantity of UA translocated into the cells at certain time periods.

### Co-immunoprecipitation detection

HEK 293 T cells underwent transfection using the designated plasmid. The Cell Lysis Buffer for immunoprecipitation was subsequently utilized to extract the proteins (Cat# P0013, Beyotime). The protein lysate was subsequently categorized into three groups: the input group, the IgG group, and the IP group. The input group was preserved at −20 °C. Rabbit IgG (Cat# 30000-0-AP, Proteintech) was incorporated into the IgG group and agitated at low speed at 4 °C for 1 h. Subsequently, HA tag antibody (Cat# 51064-2-AP, Proteintech) and Flag tag antibody (Cat# 20543-1-AP, Proteintech) were introduced to the IP group and agitated at 4 °C for 1 h. Subsequently, Protein A/G Magnetic Beads (Cat# P2080S, Beyotime) were introduced to both the IgG group and the IP group, and the samples were agitated at 4 °C overnight. The overnight samples were centrifuged and washed over five times with IP Buffer (Cat# P0013, Beyotime), followed by the addition of IP Buffer and 5 × loading buffer (Cat# P1040, Solarbio) for subsequent Western blot analysis.

### Immunofluorescence detection

The control group and TXNIP OE group of HK-2 cells underwent treatment with 4% paraformaldehyde followed by permeabilization with 0.1% Triton X-100 (Cat# GC204003, Servicebio). Subsequent to washing, cells were subjected to blocking with 5% goat serum and subsequently treated with TXNIP (Cat# ab210826, Abcam) and GLUT9 (Cat# 26486-1-AP, Proteintech) at 4 °C. Alexa Fluor 488-conjugated Goat Anti-Rabbit IgG (Cat# GB25303, Servicebio) and Alexa Fluor 594-conjugated Goat Anti-Mouse IgG (Cat# GB28303, Servicebio) were incubated for one hour, followed by a five-minute staining with DAPI (Cat# G1012, Servicebio). The slices were cleaned, prepared, and examined for fluorescence expression via fluorescence confocal microscopy.

### Statistical analysis

Data obtained from at least three replicate experiments are represented as the mean ± the standard error of the mean (SEM), and were processed and analyzed using GraphPad Prism 9. The assessment of normality and heterogeneity of variance was conducted through the utilization of the Shapiro–Wilk and Brown-Forsythe tests, respectively. The Student's t-test was employed for pair-wise comparisons, and one-way ANOVA with Tukey’s post hoc test was used for multiple group comparisons. Statistical significance was established when *P* < 0.05.

## Discussion

UA, the ultimate byproduct of purine metabolism in humans, is predominantly excreted by the kidneys and, to a lesser extent, by the intestines [5]. In healthy persons, the concentration of UA is regulated by a dynamic equilibrium between production and excretion, maintaining it at a reasonably steady level [21]. Disruptions in UA metabolism resulting from either overproduction or diminished excretion can result in hyperuricemia and gout [12, 22]. This study aims to elucidate the role of TXNIP in UA metabolism and its regulatory mechanisms in hyperuricemia and gout. TXNIP serves as a crucial regulator and enhancer in the pathogenesis of hyperuricemia and gout, significantly influencing disease progression through mechanisms including the inhibition of TRX activity, modulation of transcription factors, and the induction of oxidative stress and inflammation [23–25]. Hyperuricemia results in the deposition of urate crystals (MSU), which activates the NLRP3 inflammasome through the ROS/TXNIP/NLRP3 signaling pathway, culminating in the release of IL-1β triggered by a specific and robust inflammatory cascade response [26–28]. Furthermore, a stringent bidirectional regulatory interplay exists between TXNIP and MAPK signaling pathways in this disease [29–32]. This link not only stimulates the inflammatory response and cellular damage but also perpetuates the detrimental cycle of disrupted UA metabolism. For instance, current gout treatment drugs like colchicine partially target the MAPK pathway [33]. This aligns with our transcriptome study results, which identified a notable elevation of the MAPK signaling pathway following elevated TXNIP expression (Fig. 3C). This finding supports the notion that TXNIP plays a role in UA metabolic disorders by regulating inflammatory and cellular injury signaling pathways.

Metabolic stressors, such as elevated UA levels, have been demonstrated to produce endoplasmic reticulum stress (ERS) [34–36]. XBP1s and ATF4, crucial transcription factors in the endoplasmic reticulum stress response, can directly enhance TXNIP expression [37–39].Conversely, TXNIP may also intensify ERS (*e.g*., through ROS), establishing a positive feedback loop that perpetually exacerbates metabolic abnormalities and cellular damage signals [40]. Our current investigations validated that a high UA environment stimulated ROS formation and markedly increased TXNIP expression in HK-2 cells (Fig. 1B–D), aligning with prior findings on the UA stress response. Concurrently, the UA transporter GLUT9, responsible for reabsorption, exhibited considerable upregulation, while the UA efflux transporter ABCG2 shown significant downregulation following treatment with elevated UA levels (Fig. 1H). These data tentatively indicate that renal tubular epithelial cells demonstrate an imbalance marked by heightened UA reabsorption and diminished excretion in diseased situations.

TXNIP functions through mechanisms beyond inflammation and stress. TXNIP serves as a pivotal negative regulator of the glucose transporters GLUT1 and GLUT4, constraining cellular glucose uptake by directly inhibiting their transporter activity and membrane localization [33–36]. This mechanism not only diminishes glycolytic flux but also precipitates insulin resistance by disrupting insulin signaling pathways, including the IRS-1/PI3K/Akt cascade [37–41]. Furthermore, TXNIP triggers apoptosis and diminishes insulin release from the pancreas, consequently enhancing hepatic glucose synthesis and insulin resistance in peripheral tissues [42]. Considering the pivotal regulatory function of TXNIP in glucose and lipid metabolism [41], we hypothesize that TXNIP influences systemic UA levels by affecting UA metabolism. This study aims to investigate the potential effects of TXNIP on UA metabolism and its associated molecular mechanisms based on this hypothesis. Consequently, we performed metabolomic and transcriptome analyses. Metabolomic studies first indicated that TXNIP overexpression can influence purine metabolism (Fig. 2F). We anticipated that increased TXNIP results in aberrant synthesis of UA precursors, potentially affecting UA levels in vivo. The transcriptome findings corroborated this theory by indicating that elevated TXNIP enhances ADA expression and suppresses HPRT1 (Fig. 3D), consequently intensifying purine catabolism and facilitating UA synthesis. These results corroborated our idea. A transcriptomic study indicated that increased TXNIP expression markedly promoted the *SLC2A9* gene, responsible for encoding the UA reabsorption transporter GLUT9(Glucose transporter 9), while concurrently downregulating other ABC transporter genes. GLUT9 is a significant UA transporter protein that is crucial for proximal tubular UA reabsorption [42]. GLUT9 (*SLC2A9*), a member of the GLUT transporter family, possesses an amino acid sequence that is significantly homologous to GLUT1, indicating potential functional conservation between the two [43, 44]. Given the recognized mechanism of TXNIP interaction with GLUT1 and GLUT4, which mediates endocytosis-related degradation and signaling suppression, we hypothesized that TXNIP may regulate UA homeostasis by influencing the expression or membrane transport activity of GLUT9.ABCG2 (ATP-binding cassette sub-family G member 2) is a member of the ABC transporter family and is capable of translocating various compounds across membranes. ABCG2, a crucial UA transporter protein, influences serum UA levels by modulating UA excretion in the kidneys. Its malfunction can facilitate the onset of hyperuricemia and is pivotal in the etiology of hyperuricemia and gout [45–48]. The observed downregulation of the ABC transporter gene set suggests that TXNIP may influence UA levels by regulating UA transporter proteins. Based on this notion, we examined the expression changes of essential UA transporter proteins following TXNIP overexpression. The findings indicated that the expression of the UA reabsorption transporter GLUT9 was significantly raised in the TXNIP overexpression group (Fig. 4F), while TXNIP knockdown tests validated that this effect was selectively reversed (Fig. 5F). Simultaneously, the expression levels of TXNIP and GLUT9 were significantly elevated in HK-2 cells that stably overexpress TXNIP under high UA intervention, thereby corroborating the idea that TXNIP positively modulates GLUT9 (Fig. 5E). These findings further support the novel key regulatory role of TXNIP in the UA metabolism process.

To further investigate the relationship between TXNIP and GLUT9, we hypothesized through protein interaction analysis (Fig. 6A) that TXNIP might interact with GLUT9 to regulate UA transport in the kidney. We validated the direct protein linkage between TXNIP and its downstream target GLUT9 using Co-IP (Fig. 6B) and immunofluorescence colocalization (Fig. 6C). In summary, this study systematically investigated the regulatory role of TXNIP in UA (UA) metabolism by targeting TXNIP as a molecular marker. This was achieved through a series of experiments, including metabolomics and transcriptomics analyses, as well as the construction and validation of cellular models. The results demonstrate that TXNIP significantly modulates UA metabolism by influencing both UA synthesis and transport. This study demonstrated the involvement of the ROS-TXNIP-GLUT9 axis in renal UA metabolism at the cellular level; however, the absence of in vivo validation, such as renal-specific TXNIP knockout mice, constrains its applicability to physiological conditions. The role of inflammatory cascades triggered by excess TXNIP in the control of GLUT9 in hyperuricemia requires additional investigation. Our forthcoming research will investigate the mechanism of TXNIP in UA metabolism, encompassing its regulatory interaction with GLUT9 and the correlation between TXNIP-induced UA dysregulation and glucose/lipid metabolism, via in vivo investigations. The identification of the ROS-TXNIP-GLUT9 axis offers new therapeutic targets for hyperuricemia and gouty nephropathy, facilitating the advancement of strategies to restore UA homeostasis and mitigate metabolic diseases and complications linked to hyperuricemia and gout.

## Conclusion

This work discovered a new function of TXNIP in UA metabolism. HK-2 cells specifically stimulated ROS production in a high UA environment, leading to elevated TXNIP expression. Extensive metabolomics and transcriptomics analyses indicated that TXNIP overexpression may induce aberrant activation of purine metabolism and influence UA synthesis. In vitro tests confirmed that TXNIP knockdown restored raised GLUT9 levels due to its overexpression, leading to a reduction in net UA uptake in enhanced HK-2 cells, with this regulation mediated by the connection between TXNIP and GLUT9. The findings of this study indicate that TXNIP presents a novel therapeutic target for hyperuricemia and its related metabolic disorders.

## Supplementary Information

Supplementary material 1.

## Data Availability

The data utilized and examined in this research can be obtained from the corresponding author upon reasonable request.

## Abbreviations

UA: Uric acid
TXNIP: Thioredoxin interacting protein
ROS: Reactive oxygen species
PFK: Phosphofructokinase
G6PDH: Glucose-6-phosphate dehydrogenase
DCFH-DA: 2′,7′-Dichlorodihydrofluoresceindiacetate
GLUT9: Glucose transporter 9
ABCG2: ATP-binding cassette sub-family G member 2
URAT1: Urate transporter 1
OAT3: Organic anion transporter 3
TRX: Thioredoxin
MAPK: Mitogen-activated protein kinase
NF-κB: Nuclear factor-κB
ATP: Adenosine triphosphate
GMP: Guanosine monophosphate
ERS: Endoplasmic reticulum stress
ADA: Adenosine deaminase
HPRT1: Hypoxanthine phosphoribosyltransferase 1
XBP1s: X-box binding protein 1 spliced
ATF4: Activating transcription factor 4
IRS-1: Insulin receptor substrate 1
Pl3K: Phosphatidylinositol 3-kinase
AKT: AKT Serine/Threonine kinase
