# Supplementary material for: Molecular interplay between TXNIP and GLUT9 underlies uric acid transport dysregulation in vitro under hyperuricemic stress
Source: Eur J Med Res. 2025 Oct 9;30:942. doi: 10.1186/s40001-025-03209-8 (PMC12512431; doi:10.1186/s40001-025-03209-8)
Supplement: Supplementary file 1 — Supplementary material 1. [file 40001_2025_3209_MOESM1_ESM.docx]

**Supplementary material**

**Supplementary Table 1** The primer sequences for qPCR that were used in this study were listed.

| GENE | Primer (5’end to 3’end) |
| --- | --- |
| β-actin-F | CACCATTGGCAATGAGCGGTTC |
| β-actin-F | AGGTCTTTGCGGATGTCCACGT |
| TXNIP-F | CCGTTAGGATCCTGGCTTGC |
| TXNIP-R  GLUT9-F  GLUT9-R  URAT1-F  URAT1-R  OAT3-F  OAT3-R  ABCG2-F  ABCG2-R | GGCGCCTTGTACTCATATTTGTTTC  ACCTGCTCTTGGAGAAGCAC  CCTCTTGGGAAACGTCTGCT  CGCTTCCGACAACCTCAGTG  GGTCTCAGAGCCTGGGAATCAC  GTGGGGCTATACCGTGAAGG  TGGTCCAAAACCATCGTAACTG  GTTCTCAGCAGCTCTTCGGCTT  TCCTCCAGACACACCACGGATA |

**Supplementary Table 2** The interference target site sequences for TXNIP that were used in this study were listed.

| Target site | Sequence |
| --- | --- |
| TXNIP-oligo | CTCAAGACAGCCCTATCTTTA |
| Top strand | 5’-CACCGCTCAAGACAGCCCTATCTTTATTCAAGAGATAAAGATAGGGCTGTCTTGAGTTTTTTG-3’ |
| Bottom strand | 5’-GATCCAAAAAACTCAAGACAGCCCTATCTTTA  TCTCTTGAATAAAGATAGGGCTGTCTTGAGC-3’ |

| Structure1 | | | | Structure2 | | | | Interface area, **Å^2^** | Δ^i^G kcal/mol |
| --- | --- | --- | --- | --- | --- | --- | --- | --- | --- |
| Range | ^i^N_at_ | ^i^Nres | Surface **Å^2^** | Range | ^i^N_at_ | ^i^Nres | Surface **Å^2^** |  |  |
| C | 230 | 71 | 29240 | A | 197 | 63 | 29953 | 2130.2 | -18.7 |

**Supplementary Table 3** Protein docking results for TXNIP and GLUT9

**Supplementary Figure 1** Untargeted metabolomic analysis of HK-2 cells after TXNIP overexpression. **A:** Differential metabolite classification cycle diagram (HMDB Class); **B:** SMPDB primary pathway enrichment results.

**Supplementary Figure 2** Transcriptome analysis of HK-2 cells after TXNIP overexpression. **A:** GO function annotation results; **B:** GO function enrichment bubble chart.
